# Supplementary material for: Microbiome composition modulates the lethal outcome of Drosophila A virus infection
Source: Cell Mol Life Sci. 2026 Jan 3;83(1):61. doi: 10.1007/s00018-025-06042-8 (PMC12819904; doi:10.1007/s00018-025-06042-8)
Supplement: Supplementary file 1 [file 18_2025_6042_MOESM5_ESM.docx]

SUPPLEMENTARY MATERIAL

|  |
| --- |
| Supplementary Figure 1. A) Morphologically distinct rough colonies in L. plantarum WJL cultures that appear when plated at 30 °C. B) Quantification of rough colony prevalence and abundance in control versus Lp-supplemented flies at 12 and 20 dpi. |

|  |
| --- |
| Supplementary Figure 2. Hierarchical clustering of L. plantarum OTU 16S rDNA sequences. Multiple sequence alignment of bacterial 16S rDNA sequences was performed using MAFFT v7. Pairwise genetic distances were calculated between aligned sequences, and hierarchical clustering using average linkage was performed to visualize sequence similarity relationships among the six L. plantarum OTUs detected in fly bacterial microbiomes. Branch lengths represent genetic distance (proportion of differing nucleotide positions). |

|  |
| --- |
| Supplementary Figure 3. DAV RNA accumulation in gut and carcass tissues following oral inoculation. DAV RNA levels were measured in gut and carcass samples at 0, 2, and 7 days post-inoculation with 1000 OID of DAV and expressed as log₁₀ accumulation values. Blue bars represent non-supplemented flies and purple bars represent Lp supplemented flies. Individual data points are shown as circles (gut samples) and squares (carcass samples), with each point representing a pool of 5 individuals (n = 3 pools per group per time point). Data are presented as mean ± SE. |

|  |
| --- |
| Supplementary Figure 4. Transcriptional differences between bacterial microbiome conditions prior to viral inoculation. Volcano plot showing differential gene expression between Lp-supplemented and control (non-supplemented) flies before DAV inoculation. The x-axis represents log₂ fold change (Lp vs control) and the y-axis represents -log₁₀ adjusted p-value. Horizontal dashed line indicates significance threshold (adjusted P < 0.05) and vertical dashed lines indicate fold change thresholds (±0.5 log₂ fold change). Gray dots represent genes that do not meet significance criteria. Colored dots represent significantly differentially expressed genes, with blue indicating genes more highly expressed in control flies and purple indicating genes more highly expressed in Lp-supplemented flies. Significant genes are labeled with their names. |

|  |
| --- |
| Supplementary Figure 5. Viral variants in hosts with different bacterial microbiome. Allele frequency changes across the DAV genome comparing viral populations from control and Lp-supplemented flies at 12 days post-infection. The x-axis represents genomic position (bp) with major genomic regions indicated above, and the y-axis shows percent change of mutation frequency relative to the viral stock used as inoculum. Blue symbols indicate mutations in non-supplemented conditions while purple symbols indicate mutations in Lp-supplemented conditions. Squares represent mutations present in both conditions and triangles represent bacterial microbiome-specific mutations. Empty symbols indicate mutations already present in the inoculum and filled symbols indicate newly emerging mutations. Symbol size reflects the number of samples in which each mutation was detected. Each sample represents a pool of 5 individual flies and each condition has 4 samples. |

Supplementary Table 1. Primers used in qPCR step of RT-qPCR.

| Primer name | Sequence |
| --- | --- |
| rp49_Forward | CGGATCGATATGCTAAGCTGT |
| rp49_Reverse | GCGCTTGTTCGATCCGTA |
| DAV_Forward | GTTGGATCAGGCTAGTGTAGG |
| DAV_Reverse | TGCAACCGGACTCCAAGTTC |
| 27_Forward | AGAGTTTGATCMTGGCTCAG |
| 1391_Reverse | GACGGGCRGTGWGTRCA |

Supplementary File 1. Bacterial operational taxonomic unit (OTU) sequences and taxonomic assignments. FASTA file containing consensus sequences for all six Lactobacillus plantarum OTUs identified through 16S rDNA sequencing.

Supplementary File 2. Differentially expressed genes (DEGs) in DAV-infected flies across bacterial microbiome conditions at 12 dpe. Excel file containing differential gene expression analysis comparing DAV-infected versus mock-infected flies within non-supplemented and Lp-supplemented bacterial microbiome conditions. Mapped reads for all conditions included.

Supplementary File 3. DAV genome mutations and frequency changes from the viral stock used for inoculation. Excel file containing comprehensive analysis of viral mutations detected in DAV populations from non-supplemented and Lp-supplemented flies at 12 dpi.

Supplementary File 4. Source data for all figures. Excel file containing individual data points and measurements for all main and supplementary figures. Each worksheet corresponds to one figure panel with raw values, sample sizes, and replicate information.
